# A Gamified Smartphone App to Support Engagement in Care and Medication Adherence for HIV-Positive Young Men Who Have Sex With Men (AllyQuest): Development and Pilot Study

**DOI:** 10.2196/publichealth.8923

**Published:** 2018-04-30

**Authors:** Lisa Hightow-Weidman, Kathryn Muessig, Kelly Knudtson, Mala Srivatsa, Ellena Lawrence, Sara LeGrand, Anna Hotten, Sybil Hosek

**Affiliations:** ^1^ Institute of Global Health and Infectious Diseases University of North Carolina Chapel Hill, NC United States; ^2^ Department of Health Behavior Gillings School of Global Public Health University of North Carolina Chapel Hill, NC United States; ^3^ Ayogo Vancouver, BC Canada; ^4^ Duke Global Health Institute Duke University Durham, NC United States; ^5^ Department of Epidemiology and Biostatistics School of Public Health University of Illinois at Chicago Chicago, IL United States; ^6^ Department of Psychiatry Stroger Hospital of Cook County Chicago, IL United States

**Keywords:** YMSM, antiretroviral adherence, smartphone app, gamification, social networking

## Abstract

**Background:**

HIV disproportionately impacts young men who have sex with men (YMSM) who experience disparities across the HIV care continuum. Addressing antiretroviral therapy (ART) adherence among YMSM is an urgent public health priority. Technology-based interventions—particularly mobile health platforms—can provide tailored adherence interventions and allow YMSM to engage and connect with others.

**Objective:**

The objective of this study was to describe the development of *AllyQuest*, a novel, theoretically-based, smartphone app designed to improve engagement in care and ART adherence and social support among HIV-positive YMSM.

**Methods:**

*AllyQuest* was built on an established platform for patient engagement that embeds social networking and fundamental game mechanics, such as challenges, points, and rewards. A medication tracker provides reminders to promote ART adherence via personalized adherence strategies that are user and context specific; a calendar allows for reflection on adherence over time. After iterative development with input from two youth advisory boards, usability testing was conducted to assess app functionality, comprehension of the educational content, use of intervention features, and overall impressions of app relevance and appeal. A 28-day pilot trial was conducted with 20 HIV+ YMSM to evaluate intervention feasibility and acceptability.

**Results:**

Mean age of participants was 21.8 years (range 19-24), and 95% (19/20) of the participants were nonwhite. The mean time of app use was 158.4 min (SD 114.1), with a range of 13 to 441 min. There was a mean of 21.2 days of use (out of a total possible 28 days). There were 222 posts to the daily discussion social wall. Feasibility and acceptability ratings were high. Overall, participants found the app easy to use and navigate, not intrusive, and had few reported technical issues. Higher levels of app usage were positively correlated with HIV self-management outcomes, and there was a statistically significant (*P*<.05) positive association between the number of days logged into the app and knowledge and confidence in ability to reliably take HIV medications.

**Conclusions:**

*AllyQuest* represents a new, highly scalable solution that is well-suited to meet the specific prevention and care needs of HIV+ YMSM. The development of this intervention is both timely and vital, given the urgency of the ongoing HIV epidemic among YMSM.

## Introduction

### HIV Infection Among Young Men Who Have Sex With Men in the United States

In the United States, men who have sex with men (MSM) experience the highest rates of new HIV diagnoses, with young MSM (YMSM) and MSM of color continuing to be significantly impacted [1]. Although the number of diagnoses of HIV infection among MSM remained stable, from 2010 to 2014, the number of diagnoses among MSM in the age range of 13 to 24 years increased by 6% [2]. National HIV Behavioral Surveillance data on MSM from 20 cities found that among black MSM in the age range of 18 to 24 years tested in 2014, 26% were HIV positive, compared with 3% of white MSM. This disparity in HIV prevalence between black and white MSM increased from 2008 to 2014, especially among YMSM [3]. Young Hispanic or Latino MSM had a 20% increase in numbers of diagnoses of HIV infection from 2010-2014 [2].

HIV also disproportionately impacts YMSM across the HIV care continuum, with disparities in linkage, retention, antiretroviral therapy (ART) adherence, and viral suppression [4,5]. YMSM have documented low levels of ART adherence, impeding their likelihood of achieving viral suppression [6]. One study among 13 sites in the US Adolescent Trials Network found only 7% of diagnosed youth (81% male, 72% black, and 70% gay or bisexual) achieved viral suppression [5], which was substantially lower than the estimated 50% viral suppression for all age groups [4,5]. Alarmingly, a recent study of 991 HIV-infected YMSM (aged 15-26 years) found that 69.4% had a detectable viral load; two-thirds of whom (n=458) reported recent condomless anal sex [7].

### Barriers to Adherence Among Youth

Individual-level stated barriers to adherence among youth include forgetting, not feeling like taking medication, and not wanting to be reminded of HIV [8]. Additional factors contributing to suboptimal adherence and viral suppression among youth, including YMSM, include low medication adherence self-efficacy [9], psychological distress (depression and anxiety) [10-13], substance use (alcohol, marijuana, and other drugs) [10,13,14], structural barriers (eg, homelessness and health insurance) [15], low social support [11,16], and HIV-related stigma [10,11,17]. Multiple factors are often present, and these *syndemics* are associated with greater likelihood of nonadherence and detectable viral load in a dose-response nature [10,18] that also shows disproportionate impact on minority MSM [18].

There are only a few published interventions focused on supporting ART adherence among YMSM [19,20]. A 2017 systematic review of interventions along the HIV care continuum identified 117 medication adherence interventions. Only 9 (9/117, 8%) focused on adolescents or youth, and only 2 were specifically designed for MSM [19]. A 2014 review of adherence interventions meeting the Centers for Disease Control and Prevention’s criteria for evidence-based interventions found none that exclusively focused on MSM or youth [21]. Due to the often marginalized and stigmatized status that many YMSM living with HIV endure, there is a need for the development of tailored interventions that account for the unique and challenging circumstances they face.

Tailored interventions have been found to produce higher rates of behavior change and maintenance than nontailored programs in a variety of health domains, including HIV [22-24]. Technology-based interventions—particularly mobile health (mHealth) platforms—can provide tailored adherence interventions and allow YMSM to engage and connect with others [25-27]. mHealth tools also offer the capacity to design and deliver tailored content that best meets the specific HIV management challenges faced by YMSM and each individual end user [25]. The fidelity to intervention delivery provided by mHealth and the market saturation of mobile technology ownership across socioeconomic strata [28,30] also provides a high-impact platform that can be taken to scale across and beyond the United States. Daily mobile phone–based contact is acceptable to youth living with HIV and is associated with improved adherence [29]. In this paper, we describe the development, usability evaluation, and subsequent pilot testing of *AllyQuest*, a tailored mobile phone app to increase engagement in HIV care, ART adherence, and social support.

## Methods

### Intervention Development (September 2015-May 2016)

*AllyQuest* is a novel, theoretically-based, mobile phone app intervention designed to improve engagement in care and ART adherence and social support among HIV-positive YMSM (target age: 16-24 years). *AllyQuest* development was guided by evidence-based risk reduction and medication adherence interventions [30-32]; health behavior change theories, including social cognitive theory (SCT) [33,34]; narrative communication (eg, storytelling) [35-38]; and the principles of persuasive technology [39]. *AllyQuest* addresses key principles of SCT, including (1) Observational learning by participating in daily activities, (2) Modeling and vicarious experiences (observing and participating in daily discussions, exploration of narrative “choose-your-own-adventure” stories), (3) Self-efficacy and verbal persuasion from expert sources (multimedia knowledge center and tailored messages), and (4) Reinforcements (virtual rewards and achievements) [33,34-40]. Narrative storytelling relies on the modeling of behaviors by similar others and has been shown to foster self-reflection and influence disease self-management [41-43]. The Fogg Behavior Model (FBM) [39] of persuasive technology informed the development of Ayogo’s Empower Platform, the operating system on which *AllyQuest* was developed. According to the FBM, the principal factors to promote behavior change using technology include triggers, ability, and motivation. The design of *AllyQuest* enhances motivation and skills and provides triggers to encourage positive behaviors. App notifications are *triggers* for healthy behaviors. Regular behavioral self-report prompts serve as additional triggers and help participants establish healthy habits. *Ability* is increased through knowledge and by identifying small steps toward target behavioral goals (eg, understanding side effects and knowing how to fill a prescription). Participants also get tips from others who are dealing with similar issues and through narrative stories within the app that reinforce the consequences of healthy and unhealthy behaviors. App *motivators* include social support, rewards, goal setting, and achievements.

Concepts for *AllyQuest* design and overall “look and feel” were informed by research we conducted with HIV-positive YMSM to understand technology utilization, the barriers and facilitators to ART adherence, and the use of an app to assist with adherence [26,44,45]. Gamification elements incorporated into *AllyQuest* included the ability to “level up,” earn and redeem in-app virtual currency, and the ability to unlock app features. Throughout development, we worked closely with two YMSM youth advisory boards, composed of eight HIV-positive YMSM, located in Durham, North Carolina and Chicago, Illinois. Youth advisory board activities included 13 in-person sessions and seven rounds of Web-based surveys.

### Usability Testing (June 2016-August 2016)

Usability testing was conducted according to established usability guidelines [46] one-on-one with eight (nonyouth advisory board) HIV-positive YMSM aged 16 to 24 years. Participants were guided through app installation on their personal phones. They were asked to explore the app and complete specified tasks within the app without study staff assistance. While exploring the app, participants were asked to “think aloud” and provide a running commentary of their thought processes while performing the tasks [47]. The concurrent think aloud method was chosen to elicit real-time feedback and emotional responses [46,48,49]. Participants then received a guided tour of all app features. Participants were asked about their initial impressions of the app and completed a posttest survey to assess user experience. Participants were then asked to use the app daily for 1 week to assess ongoing functionality, monitor for any technical issues, ensure content comprehension, evaluate intervention features, and describe overall impressions of app relevance and appeal. To facilitate social connectivity, youth advisory board members also used the app during the usability testing period. A semistructured phone interview was conducted at the end of the 1-week testing period.

### Intervention Refinement (August 2016-October 2016)

The research team collated all participant feedback into a usability report that was presented to the technological partner. Usability testing revealed several addressable technical bugs and user experience issues that were resolved. Usability participants also provided specific recommendations for content edits and expansion. The research team addressed as many of these suggestions as possible before pilot launch, and the remaining suggestions were prioritized for the next iteration of development.

### Pilot Evaluation (October 2016-January 2017)

A 4-week pilot trial was then conducted with 20 HIV-positive YMSM to evaluate intervention feasibility and acceptability. Participants were recruited from a clinic in Chicago that primarily serves impoverished communities. Participants completed a pretest survey and then staff assisted with app download to participants’ phones. After 4 weeks of use, participants completed an online posttest survey and a phone-based qualitative interview.

### Pilot Trial Measures

#### Sociodemographic Items

Sociodemographic items assessed age, race or ethnicity, education, income, homelessness, health insurance, and sexual identity.

#### Feasibility

Usage data was captured through in-app analytics and included number of times per day or week participants accessed the app and average time spent using app, daily number of activities completed and daily discussion questions answered, content of posts, and number of health-focused daily quests completed.

#### Acceptability

System Usability Scale (SUS) [50] is a 10-item, 5-point Likert scale of subjective assessments of usability. The SUS provides a global measure of system satisfaction and subscales of usability and learnability. For this trial, 9 of the 10 items were used, as one question was deemed duplicative.

Client Satisfaction Questionnaire-8 (CSQ-8) was used to assess global intervention satisfaction. The CSQ-8 has eight items (quality of app, kind of service received from app, app met needs, recommend app to a friend, amount of help received from app, effectiveness of app for dealing with health problem, overall satisfaction, and willingness to use the app again). These domains are assessed on a 4-point response scale with individually specified anchors. Participant responses are scored from 1 to 4, and thus, the possible total scores range from 8 to 32. Higher scores indicate greater satisfaction. The CSQ-8 has demonstrated high internal consistency across a large number of studies and has been used to evaluate technology-based interventions [51-55].

#### HIV Self-Management

We developed four questions to assess domains of HIV-specific self-management after versus before the 1-month pilot trial. These included feeling connected to others with HIV, knowledge about HIV, ability to effectively manage HIV, and ability to reliably take ART. All outcomes were measured on a 5-point Likert scale, ranging from 1=“Much less” to 5=“Much more.”

### Pilot Trial Analysis

Frequencies and measures of central tendency (means, medians, and SDs) were calculated to describe the sample in terms of sociodemographics and acceptability, feasibility, and 4-week retention. Chi-square tests, *t* tests, and correlations were examined to provide preliminary effect estimates. Exact statistical tests were used where possible to account for the small sample size. The Spearman rank order correlation coefficient was calculated to assess the magnitude of association between app usage and HIV self-management outcomes, where a value of 0 indicates no correlation, and higher values indicate stronger association between variables [56]. The Spearman rank order correlation is a nonparametric alternative to the Pearson correlation [57] based on ranks instead of absolute values and is less sensitive to outliers and nonnormal variable distributions. Statistical analyses were conducted using SAS (SAS Institute Inc) software version 9.4 for Windows.

## Results

### Intervention Development

Youth advisory board members provided feedback on (1) Intervention structure and format (eg, organization of the intervention, appropriateness and appeal of language and images, and ease of navigation); (2) Intervention content and activities (eg, comprehension, acceptability, and relevance); and (3) Overall app impressions (eg, utility, interest, and enjoyment). Youth advisory board members’ feedback informed content development in all app components (Table 1).

**Table 1:** *AllyQuest* intervention components and scientific rationale.

Feature description		Scientific rationale
**Profile page**		
	Privacy features: **t**hese include avatars, pseudonyms, confidential pin number to open app, app time-out after 5 min of inactivity, and medication tracker that allows participants to choose any name (real or made-up) they want for their medication reminder.	Anonymity and privacy recognized as important for YMSM^a^ electronic health. Found in work done by our team and others [58,59].
	App progression meter: **v**isual display of current app “level” and in-game currency that is visible to other participants. Participants level up and earn in-game currency based on app use. Redeem currency to unlock narratives and other app features.	Game-based elements (eg, levels and competition) influence intervention engagement and impact [60].
**Daily discussion**		
	Social prompts: (eg, How do you remember your medication?) kick-off daily discussions to foster community, peer sharing, model successful behaviors, and provide reinforcement.	Social support and connection with others are important features for apps for HIV-positive YMSM [61].
**Medication tracker**		
	Medication reminder system: discreet personalized reminders and habit building solutions to promote ART^b^ adherence.	Medication reminders improve adherence, but may not be sufficient [62].
	Tailored adherence strategies: upon initial set-up, participants enter medication details, including the number of times/day and preferred time of day taken and any food restrictions. The app uses this information to provide suggestions on adherence strategies (eg, Take when I brush my teeth). Participants who are having adherence difficulties will received tailored feedback on new strategies and adherence tips.	Dynamic tailoring and unique feedback based on frequent assessments effectively promotes behavior change for many conditions, including HIV prevention and ART adherence [61,63].
**Brain builders**		
	Daily quest: actionable routine tasks help users set goals and build knowledge or skills.	Rated highly by usability and pilot participants. Gamification increases intervention engagement and impact [62,60].
	Brain games: quizzes and interactive exercises help users check knowledge and skill	
**Knowledge center**		
	Multimedia: presentation of information that includes HIV-related, safer-sex, relationships and general health and wellness. Users prompted with a reflection question after each article to apply the material to their lives. Visual shows progress toward completing each section.	Formative work of our team and others has identified that HIV+ YMSM desire information on both HIV-related issues and general health and wellness [64].
**Character-based narratives**		
	“Choose-your-own adventure” narratives feature HIV+ YMSM navigating common situations that impact care engagement and ART adherence (eg, unstable housing, substance use, and disclosure). Play through story paths allows the user to face hard choices that impact health, practice problem solving, and succeed or fail in a safe space.	Narrative communication through role modeling has been identified as facilitating health behavior change [35-38].

^a^YMSM: young men who have sex with men.

^b^ART: antiretroviral therapy.

### Usability Testing

Usability testing revealed several addressable technical bugs and user experience issues. Given the time frame and cost considerations with app development, we prioritized bug fixes and user experience issues. Although users had other suggestions for app improvement (eg, making the daily discussion more like Facebook and using different imagery in the profile area), these changes were deemed by the research team and technical partners to not likely impact pilot trial outcomes in a meaningful way and given development cost, were prioritized for a later study.

**Figure 1:**
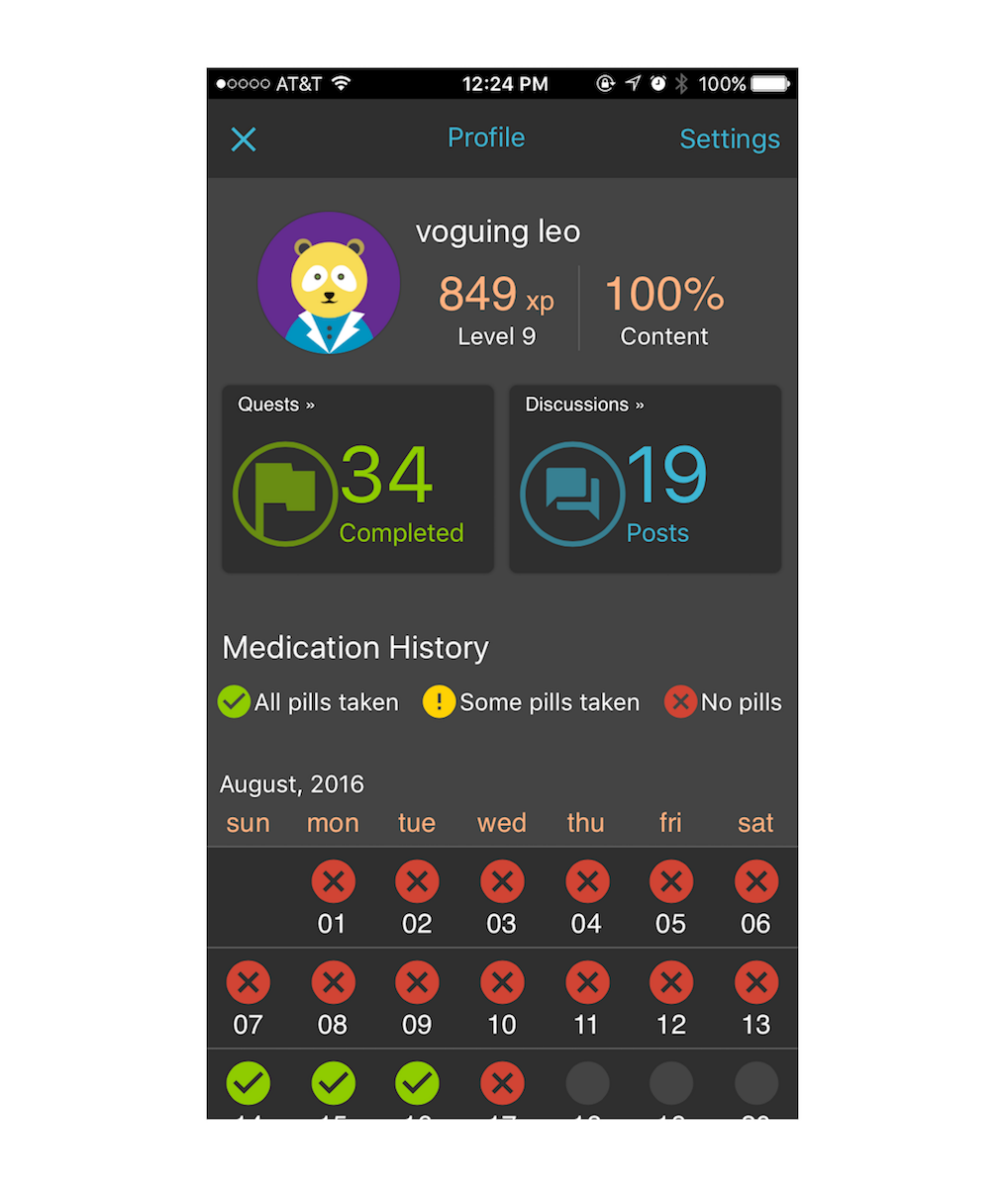
Profile page.

**Figure 2:**
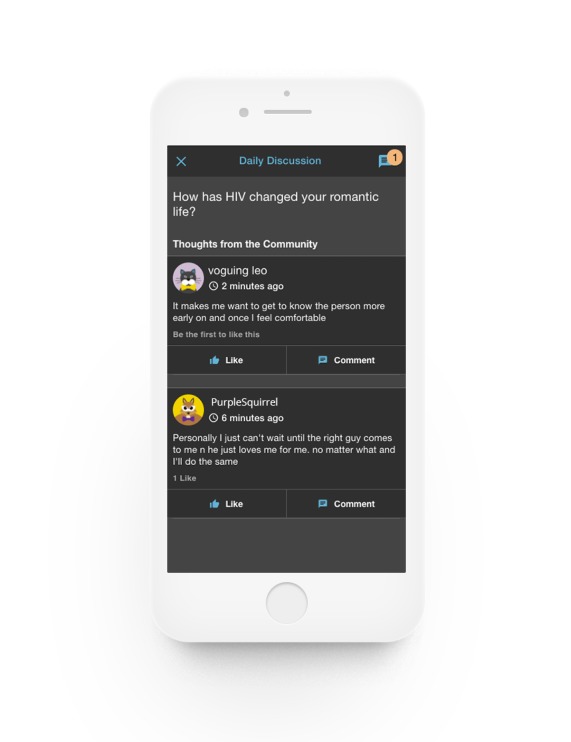
Daily discussion.

**Figure 3:**
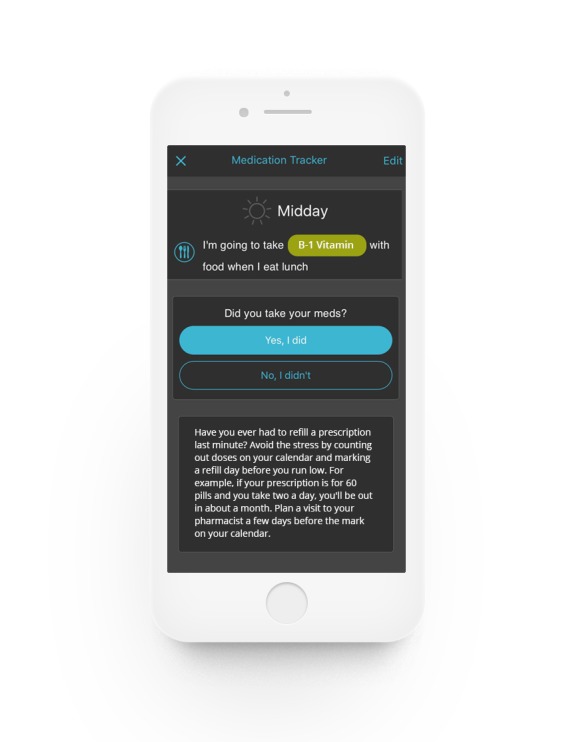
Medication tracker.

**Figure 4:**
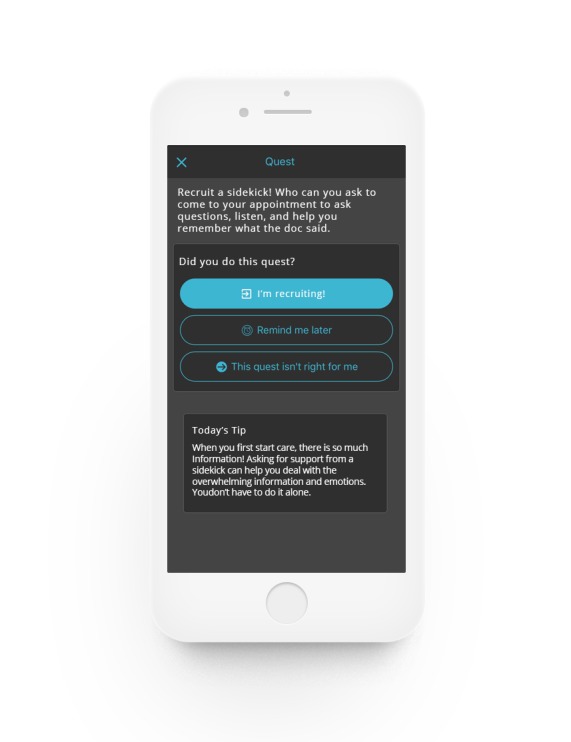
Daily quest.

**Figure 5:**
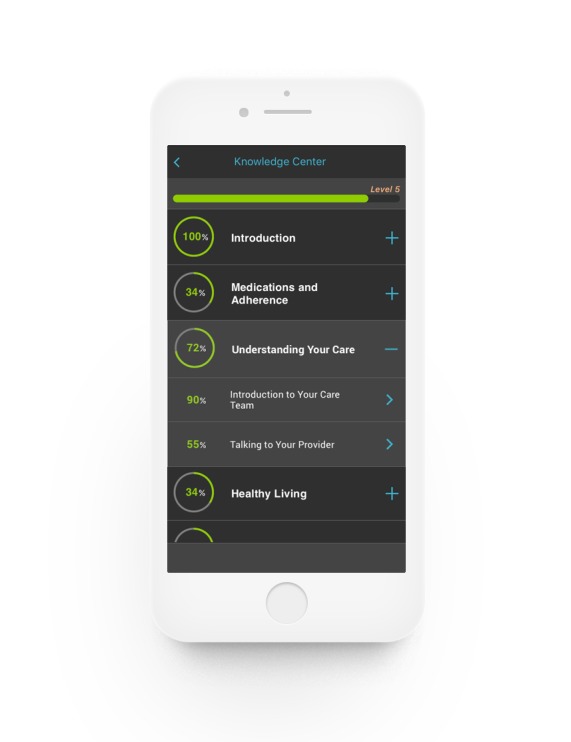
Knowledge center article.

**Figure 6:**
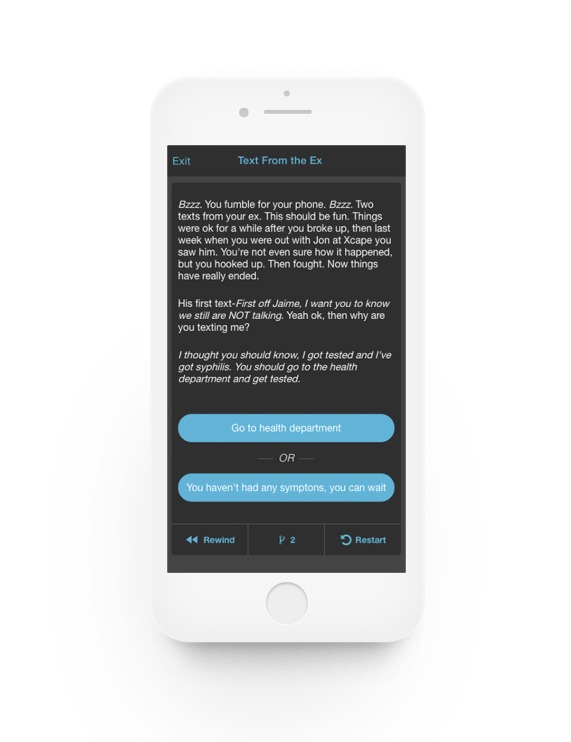
Narrative story.

App content was also reviewed by usability participants who provided specific recommendations for areas of the app that could be expanded (brain builders and daily discussion posts) and edited (collection stories and daily quests). The research team worked to address as many of these suggestions as possible before the pilot launch. Figures 1-6 provides screenshots of the fully developed *AllyQuest* app.

**Table 2:** Demographics *AllyQuest* pilot study participants, N=20.

Variable		Statistics
Age (years), mean (SD)		21.8 (1.55)
**Race or ethnicity, n (%)**		
	Black	17 (85)
	White	1 (5)
	Hispanic or Latino	1 (5)
	American Indian or Alaskan native	1 (5)
**Education, n (%)**		
	Completed high school	12 (60)
	Some college	3 (15)
	Did not complete high school	5 (25)
**Sexual identity, n (%)**		
	Gay	19 (95)
	Bisexual	1 (5)
**Employment, n (%)**		
	Currently employed	13 (65)
**Homeless last 6 months, n (%)**		
	Yes	5 (25)
**In HIV care, n (%)**		
	Yes	20 (100)
**On HIV medication, n (%)**		
	Yes	19 (95)

### Pilot

The mean age was 21.8 years (range: 19-24), 95% (19/20) were nonwhite, 95% (19/20) identified as gay, 25% (5/20) had not completed high school, 65% (13/20) were currently employed, and 25% (5/20) reported homelessness in the past 6 months (Table 2). Most (16/20, 80%) participants had been diagnosed in the past year, all reported being engaged in care, and 95% (19/20) were currently prescribed ART. One-month retention was 85%, (17/20) though all pilot participants engaged in app use during the trial.

### App Feasibility

**Figure 7:**
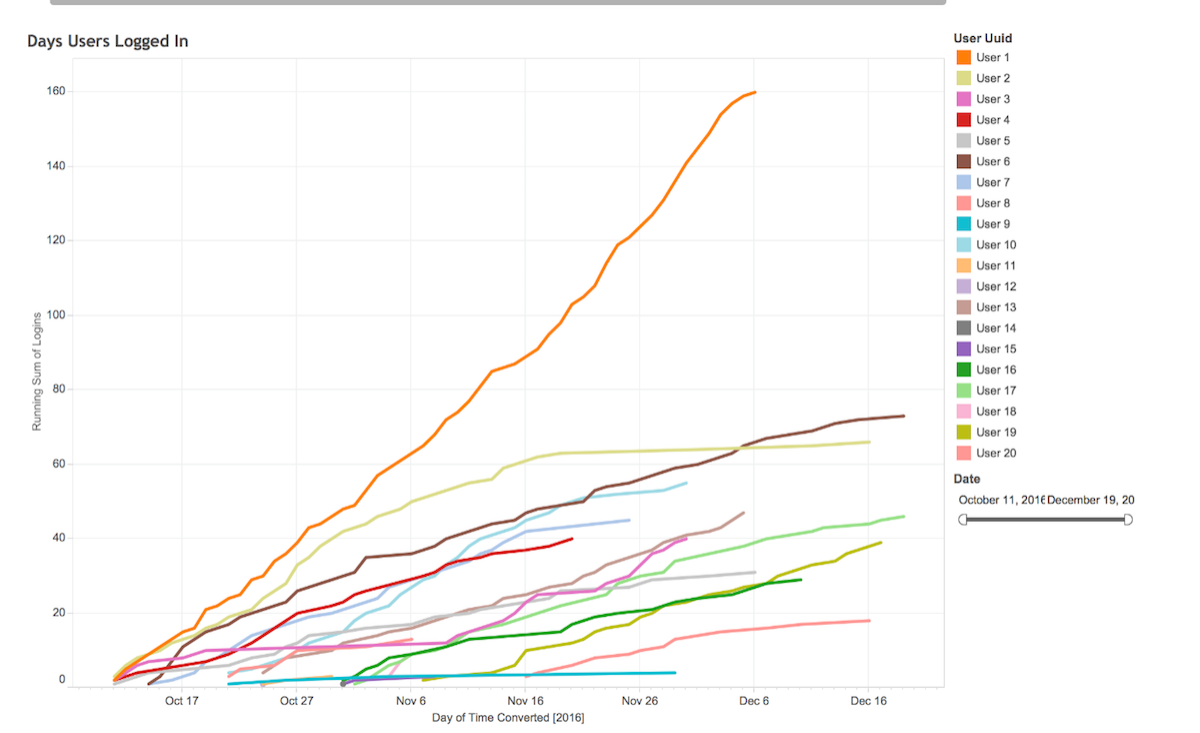
Days and log-ins among participants in *AllyQuest* pilot trial.

The mean total time of app use was 158.4 min (SD 114.1), and range was 13 to 441 min. There was a mean of 21.2 days of use with a mean of 19.4 days of logging medication (Figure 7). App usage declined over the course of the trial, with a mean of 4.3, 3.4, 3.0, and 2.8 days of usage during weeks 1, 2, 3 and 4, respectively. Although participants were told that they only needed to use the app for 4 weeks, their access to *AllyQuest* was not discontinued until the final participant completed the trial. A total of 14 participants continued to use the app after their 4-week pilot trial period ended. There was a total of 17 knowledge center articles available during the pilot. Participants read a mean of 8.3 articles (range: 0-17). A total of 45 daily discussion questions were developed by the study team that appeared to participants on consecutive days during the pilot trial (eg, a participant enrolled on October 1 would see daily discussion topic #1, whereas a participant enrolling on October 7 would see would see daily discussion topic #7). If a participant did not log on, then they would not see the daily discussion topic that day but could navigate back to those conversations to comment. There were 222 posts to the daily discussion social wall, with a median of 5 posts (range: 1-11) for each daily discussion question. Most users (16/20) posted at least once during the 4-week pilot (median: 5.5 posts/person, range: 0-41 posts). The questions that received the most posts included “What is one goal you have for your health?” (11 posts), “How do you start the safe sex conversation?” (8 posts); “How do you deal with people who react badly to your status?” (7 posts), “How did you incorporate taking meds into your routine?” (7 posts), “What qualities do you appreciate in a healthcare provider?” (7 posts,) and “How has HIV changed your romantic life?” (7 posts).

### App Acceptability

Acceptability ratings were high. Overall, participants found the app easy to use and navigate, not intrusive, and few reported technical issues (Table 3). The mean score on the CSQ-8 was 27.8 (SD 5.9). Most rated the quality of the app as excellent (n=10) or good (n=7), and overall, 15/17 were satisfied with the app. Overall, 16/17 participants felt they were getting the kind of service they wanted from the app, 14/17 felt that the app met most or almost all of their needs, 15/17 reported being mostly or very satisfied with the amount of help they received from the app, and 15/17 felt the app helped them deal more effectively with their HIV. Most (16/17) would recommend the app to a friend if they were in need of similar help, and 16/17 would use the app again.

**Table 3:** *AllyQuest* pilot study outcomes (n=17); 5-point Likert scale (1=strongly disagree-5=strongly agree).

Survey item	Mean (SD)	Median (Q1, Q3)	Minimum, maximum
Would use this app frequently	4.41 (1.18)	5.00 (4.00, 5.00)	1.00, 5.00
App was easy to use	4.76 (0.56)	5.00 (5.00, 5.00)	3.00, 5.00
Felt very confident using the app	4.47 (1.01)	5.00 (4.00, 5.00)	2.00, 5.00
App is accurate	4.53 (1.01)	5.00 (4.00, 5.00)	1.00, 5.00
App is dependable	4.53 (0.62)	5.00 (4.00, 5.00)	3.00, 5.00
Interaction with app is consistent	4.18 (0.95)	4.00 (4.00, 5.00)	2.00, 5.00
Found app unnecessarily complex	1.65 (0.86)	1.00 (1.00, 2.00)	1.00, 3.00
Would need technical assistance to use app	1.71 (1.16)	1.00 (1.00, 2.00)	1.00, 5.00
App features are well integrated	4.53 (0.80)	5.00 (4.00, 5.00)	2.00, 5.00
Most people could learn to use app quickly	4.65 (0.61)	5.00 (4.00, 5.00)	3.00, 5.00
Found app cumbersome to use	2.76 (1.64)	3.00 (1.00, 4.00)	1.00, 5.00
After versus before: connected to others with HIV	4.12 (0.70)	4.00 (4.00, 5.00)	3.00, 5.00
After versus before: ability to manage HIV	4.47 (0.72)	5.00 (4.00, 5.00)	3.00, 5.00
After versus before: ability to reliably take ART^a^	4.59 (0.71)	5.00 (4.00, 5.00)	3.00, 5.00
After versus before: knowledgeable about HIV	4.29 (0.77)	4.00 (4.00, 5.00)	3.00, 5.00

^a^ART: antiretroviral therapy.

**Table 4:** Correlations between app usage and HIV self-management outcomes, n=17. Outcome compared with before participating in the study.

App analytics	Connected to others with HIV, rho (*P* value)	Knowledgeable about condition, rho (*P* value)	Ability to manage condition, rho (*P* value)	Ability to reliably take medication, rho (*P* value)
Days logged in	.31 (.22)	.53 (.03)	.33 (.19)	.49 (.05)
Days logged medication	.34 (.20)	.42 (.10)	.19 (.48)	.41 (.11)
Total time on app	.13 (.61)	.42 (.09)	.04 (.86)	.15 (.58)
Articles read	.01 (.98)	.29 (.28)	−.10 (.72)	−.07 (.78)
Social wall posts	.34 (.18)	.48 (.52)	.40 (.12)	.35 (.17)
Daily quests	.32 (.22)	.43 (.88)	.12 (.63)	.36 (.16)

Qualitative exit interviews (n=17) identified areas for app improvement, including the need for additional tailoring and personalization. Overall, *AllyQuest* met the needs and expectations of its users, as expressed by some users:

But, you know, it did help me to overall accept that I have to take this prescription because it’s only going to help me in the long run. So it was good as far as helping me remember to take the medicine.

Participant 1004

Being that I’m not much of a public speaker, in a sense, or an extroverted personality, the app kinda allowed me to, you know, bolster my genuine personal personality in a discrete manner, it was enjoyable.

Participant 1008

Next I know I was just on this thing all day. My friends are like, “You’re always on your phone.” I’m like, “Oh yeah, it’s this app. I really can’t talk about it’s my personal life.” They’re like, “Oh, okay.” So I’m just always on my phone.

Participant 1007

The daily discussion and medication tracker were users’ favorite features. The discussions gave users a safe space to give and receive advice and made them feel less alone:

When I would read other people’s comments on the little discussion panel thing—I would feel like I wasn’t so alone...it made me feel like I was a part of a community that understood how hard it is dealing with something like that, because HIV is, like—it’s something that’s serious, and I always thought it was a death sentence because when I first found out I had it, I literally thought I was gonna die.

Participant 1020

You’re talking to profiles and it’s really, really cool cause I get a lot of feedback that I would need in my life because I don’t talk about it with a lot of people. So this is a way that I get to keep it private and to myself and also get help. So it’s really been amazing.

Participant 1007

The medication tracker helped users establish a medication strategy and normalize taking medications daily, as described by one user:

I’ve downloaded other apps where like a medicine tracker...it was just a very basic thing. And also I’ve had discussion forums on my phone where it just dove clean into something that was, like, I’m not gonna talk about that just yet. So I think the app was, like, right there in the middle. It offered the avenue to go deeper into the conversation and it offered just a safe space at the same time.

Participant 1008

Overall, users liked and trusted the health information on the app. Overall, they felt that the tone and content in the narratives was important and relevant. One participant described how these stories influenced his relationship with his partner:

Interviewer: How did the stories motivate you in the app?

Participant: It kinda motivated me to be more open with my partner...I would keep a lotta things from them. Like, I would go through stressful times and I wouldn’t tell them...but after seeing how a lotta that stuff played out, like in the [app] stories, I try to start opening up more, and I think we’re gonna stay together for a long time.

Participant 1002

Participants did comment that they wanted more information regarding sexual health, relationships, mental health, and nutrition.

### HIV Self-Management

Higher levels of app usage were positively correlated with HIV self-management outcomes, and there was a statistically significant (*P*<.05) positive association between the number of days logged into the app and knowledge and confidence in the ability to reliably take HIV medication (Table 4). Although statistical power was limited because of the small sample size, results are promising for a full intervention trial.

## Discussion

### Principal Findings

In this paper, we describe the development of a novel, theory-based ART adherence app for YMSM that showed strong acceptability, feasibility, and preliminary impact on HIV self-management outcomes. Prior work has established that technology-based interventions—particularly mHealth platforms—can provide tailored adherence interventions and allow YMSM to engage and connect with others [25-27]. Furthermore, daily mobile phone–based contact is acceptable to youth living with HIV and is associated with improved adherence [29]. Although several mHealth adherence studies for MSM are underway or under development [65,66], to our knowledge, there are no currently available interventions for YMSM that include the range of features incorporated in *AllyQuest* or are poised to utilize technology in a similar highly innovative and engaging way.

HIV-positive YMSM were instrumental in informing all stages of *AllyQuest* development. Prior research with HIV-positive YMSM informed the inclusion of essential elements in the initial *AllyQuest* prototype, including information on both HIV-related issues and general health and wellness [64], anonymity and privacy features [58,59,64], medication reminders and tailored adherence strategies [59,63,67-69], and provision of social support and a connection with others [61,70]. Youth advisory board and usability participants provided further feedback that was incorporated in an agile way to allow for ongoing modifications and enhancements. The result of this process is a user-centered, highly engaging, multicomponent care support app.

*AllyQuest* accommodates different learning styles, motivations, and needs among YMSM through features including app-guided tailoring of content, personalized messages, and inclusion of game-based elements. Gamification uses game design components in nontraditional gaming contexts, thus providing opportunities for a greater level of engagement of participants in online behavioral interventions [60,71]. Interventions can utilize gamification to deliver highly engaging content and promote participant interactions both within and outside the intervention, thus increasing the potential for health behavior change. To optimize intervention engagement and impact, *AllyQuest* integrates health-related challenges, rewards, social connectivity, and “unlocking” character-driven narratives [60,71].

*AllyQuest* was built on an established platform developed by our technology partner, Ayogo. In a world of ever-shrinking resources, developing apps for ART adherence *de novo* may not take advantage of prior work done to improve treatment adherence in other disease states, thus failing to capitalize on lessons learned and software assets developed. Furthermore, collaborating with technology partners with established products allows for some degree of cost-sharing, ensuring that the app will be updated as needed and can be scaled up if proven effective. However, this process still requires active engagement with the target population to ensure adaptations are both developmentally and culturally congruent with their needs. Allowing sufficient time for iterative adjustments to the intervention is critical.

Understanding the full spectrum of app feasibility and acceptability before large-scale efficacy testing is essential. This entails measuring and subsequently aligning multiple streams of both in app (metrics of use and participant postings) and out of app (pre- and posttest surveys and qualitative exit interviews) data. Ensuring the app has a robust back-end data system to capture all app analytics is a crucial piece that should be prioritized early in development. In this study, feasibility and acceptability metrics aligned with our primary HIV self-management outcomes, increasing the likelihood that *AllyQuest* t could in fact impact long-term HIV ART adherence among HIV-positive YMSM. However, identifying additional strategies to ensure consistent and sustained app engagement should be considered. Integration of features that provide users who may not respond to technology-only solutions (eg, two-way text messaging or video counseling sessions with an adherence counselor) or stepped transition to in-person interventions should be considered in future studies.

### Limitations

This study is not without limitations. Data on HIV self-management outcomes were self-reported and were only measured among participants who received the intervention. Due to the limited scope and length of this pilot study, enrolling a control group would not have been feasible, and measuring changes in biologic outcomes would not have been clinically meaningful. Statistical tests should be interpreted with caution, given the sample size. Findings may not be generalizable to YMSM from other sociodemographic backgrounds or geographic locations. Although we attempted to enroll diverse youth, the majority of participants were YMSM of color. Given the disproportionate impact of HIV among YMSM of color in the United States, these youth represent the population in highest need of interventions. Finally, we had 3 participants who did not complete their follow-up survey or interview, though all 3 participants logged on to the app and used it during the pilot study. Additional engagement and retention strategies will be important particularly when evaluating the impact of the app on out-of-care youth.

### Conclusions

This small pilot trial confirmed that an interactive app is feasible and acceptable to YMSM as a tool to address ART adherence. Future work should build on the promising data from this trial to test *AllyQuest* in a larger, diverse sample to assess intervention efficacy for improving ART adherence and increasing sustained viral suppression. If a highly scalable technology such as *AllyQuest* could ultimately demonstrate effectiveness in implementation studies, it would be a powerful tool for realizing the individual and public health benefits of biomedical advances in prevention and care therapies. Furthermore, placing these tailored technologies in the hands of YMSM offers an approach to HIV self-management that may empower youth as they establish optimal HIV care engagement habits for the future.

## Abbreviations

ART: antiretroviral therapy
CSQ-8: Client Satisfaction Questionnaire-8
FBM: Fogg Behavior Model
mHealth: mobile health
MSM: men who have sex with men
SCT: social cognitive theory
SUS: System Usability Scale
YMSM: young men who have sex with men

## References

[1] Centers for Disease Control and Prevention (2016). https://www.cdc.gov/hiv/library/reports/hiv-surveillance.html.

[2] Centers for Disease Control and Prevention (2015). https://www.cdc.gov/hiv/pdf/library/reports/surveillance/cdc-hiv-surveillance-report-2015-vol-27.pdf.

[3] Wejnert C, Hess KL, Rose CE, Balaji A, Smith JC, Paz-Bailey G, NHBS Study Group (2016). Age-Specific Race and Ethnicity Disparities in HIV Infection and Awareness Among Men Who Have Sex With Men--20 US Cities, 2008-2014. J Infect Dis.

[4] Centers for Disease Control and Prevention (2015). https://www.cdc.gov/hiv/pdf/library/reports/surveillance/cdc-hiv-surveillance-supplemental-report-vol-22-2.pdf.

[5] Kapogiannis BX, Mayer K The HIV continuum of care for adolescents and young adults (12-24 years) attending 13 urban US centers of the NICHD-ATN-CDC-HRSA SMILE collaborative.

[6] Kim SH, Gerver SM, Fidler S, Ward H (2014). Adherence to antiretroviral therapy in adolescents living with HIV: systematic review and meta-analysis. AIDS.

[7] Wilson PA, Kahana SY, Fernandez MI, Harper GW, Mayer K, Wilson CM, Hightow-Weidman LB (2016). Sexual risk behavior among virologically detectable human immunodeficiency virus-infected young men who have sex with men. JAMA Pediatr.

[8] MacDonell K, Naar-King S, Huszti H, Belzer M (2013). Barriers to medication adherence in behaviorally and perinatally infected youth living with HIV. AIDS Behav.

[9] Houston E, Fominaya AW (2015). Antiretroviral therapy adherence in a sample of men with low socioeconomic status: the role of task-specific treatment self-efficacy. Psychol Health Med.

[10] Kuhns LM, Hotton AL, Garofalo R, Muldoon AL, Jaffe K, Bouris A, Voisin D, Schneider J (2016). An index of multiple psychosocial, syndemic conditions is associated with antiretroviral medication adherence among HIV-positive youth. AIDS Patient Care STDS.

[11] Murphy DA, Marelich WD, Hoffman D, Steers WN (2004). Predictors of antiretroviral adherence. AIDS Care.

[12] Shacham E, Estlund AL, Tanner AE, Presti R (2017). Challenges to HIV management among youth engaged in HIV care. AIDS Care.

[13] White JM, Gordon JR, Mimiaga MJ (2014). The role of substance use and mental health problems in medication adherence among HIV-infected MSM. LGBT Health.

[14] Gross IM, Hosek S, Richards MH, Fernandez MI (2016). Predictors and profiles of antiretroviral therapy adherence among African American adolescents and young adult males living with HIV. AIDS Patient Care STDS.

[15] Rudy BJ, Murphy DA, Harris DR, Muenz L, Ellen J, Adolescent Trials Network for HIV/AIDS Interventions (2009). Patient-related risks for nonadherence to antiretroviral therapy among HIV-infected youth in the United States: a study of prevalence and interactions. AIDS Patient Care STDS.

[16] Macdonell KE, Naar-King S, Murphy DA, Parsons JT, Harper GW (2010). Predictors of medication adherence in high risk youth of color living with HIV. J Pediatr Psychol.

[17] Bogart LM, Landrine H, Galvan FH, Wagner GJ, Klein DJ (2013). Perceived discrimination and physical health among HIV-positive Black and Latino men who have sex with men. AIDS Behav.

[18] Friedman MR, Stall R, Silvestre AJ, Wei C, Shoptaw S, Herrick A, Surkan PJ, Teplin L, Plankey MW (2015). Effects of syndemics on HIV viral load and medication adherence in the multicentre AIDS cohort study. AIDS.

[19] Risher KA, Kapoor S, Daramola AM, Paz-Bailey G, Skarbinski J, Doyle K, Shearer K, Dowdy D, Rosenberg E, Sullivan P, Shah M (2017). Challenges in the evaluation of interventions to improve engagement along the HIV care continuum in the United States: a systematic review. AIDS Behav.

[20] Chaiyachati KH, Ogbuoji O, Price M, Suthar AB, Negussie EK, Bärnighausen T (2014). Interventions to improve adherence to antiretroviral therapy: a rapid systematic review. AIDS.

[21] Charania MR, Marshall KJ, Lyles CM, Crepaz N, Kay LS, Koenig LJ, Weidle PJ, Purcell DW, HIV/AIDS Prevention Research Synthesis (PRS) Team (2014). Identification of evidence-based interventions for promoting HIV medication adherence: findings from a systematic review of U.S.-based studies, 1996-2011. AIDS Behav.

[22] Noar SM, Benac CN, Harris MS (2007). Does tailoring matter? Meta-analytic review of tailored print health behavior change interventions. Psychol Bull.

[23] Kreuter MW, Wray RJ (2003). Tailored and targeted health communication: strategies for enhancing information relevance. Am J Health Behav.

[24] Krebs P, Prochaska JO, Rossi JS (2010). A meta-analysis of computer-tailored interventions for health behavior change. Prev Med.

[25] Hightow-Weidman LB, Muessig KE, Bauermeister J, Zhang C, LeGrand S (2015). Youth, technology, and HIV: recent advances and future directions. Curr HIV/AIDS Rep.

[26] LeGrand S, Muessig KE, McNulty T, Soni K, Knudtson K, Lemann A, Nwoko N, Hightow-Weidman LB (2016). Epic Allies: development of a gaming app to improve antiretroviral therapy adherence among young HIV-positive men who have sex with men. JMIR Serious Games.

[27] Muessig KE, Nekkanti M, Bauermeister J, Bull S, Hightow-Weidman LB (2015). A systematic review of recent smartphone, Internet and Web 2.0 interventions to address the HIV continuum of care. Curr HIV/AIDS Rep.

[28] Shaw RJ, Steinberg DM, Zullig LL, Bosworth HB, Johnson CM, Davis LL (2014). mHealth interventions for weight loss: a guide for achieving treatment fidelity. J Am Med Inform Assoc.

[29] Belzer ME, Kolmodin MacDonell K, Clark LF, Huang J, Olson J, Kahana SY, Naar S, Sarr M, Thornton S, Adolescent Medicine Trials Network for HIV/AIDS Interventions (2015). Acceptability and feasibility of a cell phone support intervention for youth living with HIV with nonadherence to antiretroviral therapy. AIDS Patient Care STDS.

[30] Garofalo R, Kuhns LM, Hotton A, Johnson A, Muldoon A, Rice D (2016). A randomized controlled trial of personalized text message reminders to promote medication adherence among HIV-positive adolescents and young adults. AIDS Behav.

[31] Johnson MO, Charlebois E, Morin SF, Remien RH, Chesney MA, National Institute of Mental Health Healthy Living Project Team (2007). Effects of a behavioral intervention on antiretroviral medication adherence among people living with HIV: the healthy living project randomized controlled study. J Acquir Immune Defic Syndr.

[32] Wilton L, Herbst JH, Coury-Doniger P, Painter TM, English G, Alvarez ME, Scahill M, Roberson MA, Lucas B, Johnson WD, Carey JW (2009). Efficacy of an HIV/STI prevention intervention for black men who have sex with men: findings from the Many Men, Many Voices (3MV) project. AIDS Behav.

[33] Bandura A (2004). Health promotion by social cognitive means. Health Educ Behav.

[34] Bandura A (1989). Human agency in social cognitive theory. Am Psychol.

[35] Fix GM, Houston TK, Barker AM, Wexler L, Cook N, Volkman JE, Bokhour BG (2012). A novel process for integrating patient stories into patient education interventions: incorporating lessons from theater arts. Patient Educ Couns.

[36] Houston TK, Cherrington A, Coley HL, Robinson KM, Trobaugh JA, Williams JH, Foster PH, Ford DE, Gerber BS, Shewchuk RM, Allison JJ (2011). The art and science of patient storytelling-harnessing narrative communication for behavioral interventions: the ACCE project. J Health Commun.

[37] Petraglia J (2007). Narrative intervention in behavior and public health. J Health Commun.

[38] Petraglia J, Galavotti C, Harford N, Pappas-DeLuca KA, Mooki M (2007). Applying behavioral science to behavior change communication: the pathways to change tools. Health Promot Pract.

[39] Fogg BJ (2002). Persuasive technology: using computers to change what we think and do. Ubiquity.

[40] Abraham C, Sheeran P, Orbell S (1998). Can social cognitive models contribute to the effectiveness of HIV-preventive behavioural interventions? A brief review of the literature and a reply to Joffe (1996; 1997) and Fife-Schaw (1997). Br J Med Psychol.

[41] Gucciardi E, Jean-Pierre N, Karam G, Sidani S (2016). Designing and delivering facilitated storytelling interventions for chronic disease self-management: a scoping review. BMC Health Serv Res.

[42] Houston TK, Allison JJ, Sussman M, Horn W, Holt CL, Trobaugh J, Salas M, Pisu M, Cuffee YL, Larkin D, Person SD, Barton B, Kiefe CI, Hullett S (2011). Culturally appropriate storytelling to improve blood pressure: a randomized trial. Ann Intern Med.

[43] McQueen A, Kreuter MW, Kalesan B, Alcaraz KI (2011). Understanding narrative effects: the impact of breast cancer survivor stories on message processing, attitudes, and beliefs among African American women. Health Psychol.

[44] Hightow-Weidman LB, Muessig K, Srivatsa M, Lawrence E, LeGrand S, Kirschke-Schwartz H, Lemos D, Balthazar C, Hosek S AllyQuest: Engaging HIV Young MSM in Care and Improving Adherence through a Social Networking and Gamified Smartphone App.

[45] Hightow-Weidman LB, Muessig KE, Pike EC, LeGrand S, Baltierra N, Rucker AJ, Wilson P (2015). HealthMpowerment.org: building community through a mobile-optimized, online health promotion intervention. Health Educ Behav.

[46] Usability https://www.usability.gov/.

[47] Rubin J, Chisnell D (2008). Handbook of Usability Testing: How to Plan, Design, and Conduct Effective Tests Second Edition.

[48] Olmsted-Hawala EL, Murphy ED, Hawala S, Ashenfelter KT Does age make a difference?.

[49] Richardson S, Mishuris R, O'Connell A, Feldstein D, Hess R, Smith P, McCullagh L, McGinn T, Mann D (2017). “Think aloud” and “Near live” usability testing of two complex clinical decision support tools. Int J Med Inform.

[50] Brooke J, Weerdmester B, McClelland I (1996). A quick and dirty usability scale. Usability Evaluation in Industry.

[51] Matsubara C, Green J, Astorga LT, Daya EL, Jervoso HC, Gonzaga EM, Jimba M (2013). Reliability tests and validation tests of the client satisfaction questionnaire (CSQ-8) as an index of satisfaction with childbirth-related care among Filipino women. BMC Pregnancy Childbirth.

[52] Cordova D, Alers-Rojas F, Lua FM, Bauermeister J, Nurenberg R, Ovadje L, Fessler K, Delva J, Salas-Wright CP, Council YL (2016). The usability and acceptability of an Aaolescent mHealth HIV/STI and drug abuse preventive intervention in primary care. Behav Med.

[53] Tsai LLY, McNamara RJ, Dennis SM, Moddel C, Alison JA, McKenzie DK, McKeough ZJ (2016). Satisfaction and experience with a supervised home-based real-time videoconferencing telerehabilitation exercise program in people with chronic obstructive pulmonary disease (COPD). Int J Telerehabil.

[54] Nobis S, Lehr D, Ebert DD, Baumeister H, Snoek F, Riper H, Berking M (2015). Efficacy of a web-based intervention with mobile phone support in treating depressive symptoms in adults with type 1 and type 2 diabetes: a randomized controlled trial. Diabetes Care.

[55] Donker T, Bennett K, Bennett A, Mackinnon A, van Straten A, Cuijpers P, Christensen H, Griffiths KM (2013). Internet-delivered interpersonal psychotherapy versus internet-delivered cognitive behavioral therapy for adults with depressive symptoms: randomized controlled noninferiority trial. J Med Internet Res.

[56] Astivia OL, Zumbo BD (2017). Population models and simulation methods: the case of the Spearman rank correlation. Br J Math Stat Psychol.

[57] Janosky JE (1991). Pearson correlation coefficients vs reliability coefficients. J Am Diet Assoc.

[58] Muessig KE, Pike EC, Fowler B, LeGrand S, Parsons JT, Bull SS, Wilson PA, Wohl DA, Hightow-Weidman LB (2013). Putting prevention in their pockets: developing mobile phone-based HIV interventions for black men who have sex with men. AIDS Patient Care STDS.

[59] Senn TE, Braksmajer A, Coury-Doniger P, Urban MA, Carey MP (2017). Mobile technology use and desired technology-based intervention characteristics among HIV+ black men who have sex with men. AIDS Care.

[60] Cugelman B (2013). Gamification: what it is and why it matters to digital health behavior change developers. JMIR Serious Games.

[61] Senn TE, Braksmajer A, Coury-Doniger P, Urban MA, Rossi A, Carey MP (2017). Development and preliminary pilot testing of a peer support text messaging intervention for HIV-infected black men who have sex with men. J Acquir Immune Defic Syndr.

[62] Saberi P, Johnson MO (2011). Technology-based self-care methods of improving antiretroviral adherence: a systematic review. PLoS One.

[63] Lewis MA, Uhrig JD, Bann CM, Harris JL, Furberg RD, Coomes C, Kuhns LM (2013). Tailored text messaging intervention for HIV adherence: a proof-of-concept study. Health Psychol.

[64] Saberi P, Siedle-Khan R, Sheon N, Lightfoot M (2016). The use of mobile health applications among youth and young adults living with HIV: focus group findings. AIDS Patient Care STDS.

[65] Tanner AE, Mann L, Song E, Alonzo J, Schafer K, Arellano E, Garcia JM, Rhodes SD (2016). weCARE: a social media-based intervention designed to increase HIV care linkage, retention, and health outcomes for racially and ethnically diverse young MSM. AIDS Educ Prev.

[66] Muessig KE, LeGrand S, Horvath KJ, Bauermeister JA, Hightow-Weidman LB (2017). Recent mobile health interventions to support medication adherence among HIV-positive MSM. Curr Opin HIV AIDS.

[67] Dowshen N, Kuhns LM, Johnson A, Holoyda BJ, Garofalo R (2012). Improving adherence to antiretroviral therapy for youth living with HIV/AIDS: a pilot study using personalized, interactive, daily text message reminders. J Med Internet Res.

[68] Schnall R, Bakken S, Rojas M, Travers J, Carballo-Dieguez A (2015). mHealth technology as a persuasive tool for treatment, care and management of persons living with HIV. AIDS Behav.

[69] Schnall R, Mosley JP, Iribarren SJ, Bakken S, Carballo-Diéguez A, Brown IW (2015). Comparison of a user-centered design, self-management app to existing mHealth apps for persons living with HIV. JMIR Mhealth Uhealth.

[70] Horvath KJ, Alemu D, Danh T, Baker JV, Carrico AW (2016). Creating effective mobile phone apps to optimize antiretroviral therapy adherence: perspectives from stimulant-using HIV-positive men who have sex with men. JMIR Mhealth Uhealth.

[71] King D, Greaves F, Exeter C, Darzi A (2013). 'Gamification': influencing health behaviours with games. J R Soc Med.

